# An Exploratory Association Analysis of *ABCB*_1_ rs1045642 and *ABCB*_1_ rs4148738 with Non-Major Bleeding Risk in Atrial Fibrillation Patients Treated with Dabigatran or Apixaban

**DOI:** 10.3390/jpm10030133

**Published:** 2020-09-18

**Authors:** Adela-Nicoleta Roşian, Mihaela Iancu, Adrian Pavel Trifa, Ştefan Horia Roşian, Cristina Mada, Cornelia Paula Gocan, Teodora Niţă, Sabina Istratoaie, Paul-Mihai Boarescu, Anca Dana Buzoianu

**Affiliations:** 1 Department of Pharmacology, Toxicology and Clinical Pharmacology, “Iuliu Haţieganu” University of Medicine and Pharmacy Cluj-Napoca, 23 Gheorghe Marinescu Street, 400337 Cluj-Napoca, Romania; adelarosianu@gmail.com (A.-N.R.); sabina.istratoaie@gmail.com (S.I.); abuzoianu@umfcluj.ro (A.D.B.); 2 “Niculae Stăncioiu” Heart Institute Cluj-Napoca, 19-21 Calea Moților Street, 400001 Cluj-Napoca, Romania; cristina.mada12@gmail.com (C.M.); paula.gocan@gmail.com (C.P.G.); teonita82@gmail.com (T.N.); 3 Department of Medical Informatics and Biostatistics, “Iuliu Hațieganu” University of Medicine and Pharmacy, 6 Louis Pasteur, 400349 Cluj-Napoca, Romania; miancu@umfcluj.ro; 4 Department of Genetics, “Iuliu Haţieganu” University of Medicine and Pharmacy Cluj-Napoca, 6 Louis Pasteur Street, 400349 Cluj-Napoca, Romania; trifa.adrian@gmail.com; 5 Department of Cardiology—Heart Institute, “Iuliu Haţieganu” University of Medicine and Pharmacy Cluj-Napoca, 19-21 Calea Moților Street, 400001 Cluj-Napoca, Romania; 6 Department of Pathophysiology, Iuliu Haţieganu University of Medicine and Pharmacy Cluj-Napoca, 2-4 Victor Babeş Street, 400012 Cluj-Napoca, Romania; boarescu.paul@umfcluj.ro

**Keywords:** *ABCB*_1_, apixaban, bleedings, clinical practice, dabigatran, pharmacogenomics

## Abstract

(1) Background: The approach of bleeding complications in patients treated with non-vitamin K oral anticoagulants (NOACs) represents an important issue in clinical practice. Both dabigatran and apixaban are substrates for P-glycoprotein and, therefore, *ABCB*_1_ gene variations may be useful in individualizing NOACs treatment, especially in high-risk patients. (2) Methods: *ABCB*_1_ rs1045642 and rs4148738 were determined in 218 atrial fibrillation patients treated with dabigatran or apixaban (70.94 ± 9.04 years; 51.83% men). (3) Results: Non-major bleeding appeared in 7.34% NOACs–treated patients. The logistic tested models based on the four genetic models revealed no significant association between the variant genotype of two *ABCB*_1_ SNPs and the risk of bleeding (*p* > 0.05). Among the four two-locus haplotypes, TA and CA haplotypes had the highest frequency in NOACs-treated patients with bleeding, involving a possible positive association with the susceptibility of bleeding complications (OR = 1.04 and OR = 1.91, respectively). The logistic model found no significant association of estimated haplotypes with bleeding (*p* > 0.05) except for the TG haplotype which had a trend toward statistical significance (*p* = 0.092). Among the risk factors for bleeding, only age > 70 years and stroke/TIA showed a tendency toward statistical significance. (4) Conclusions: We found no significant associations between the studied *ABCB*_1_ variant genotypes with non-major bleeding risk in NOACs-treated patients. A trend of association between TG haplotype with bleeding risk was observed, implying a protective role of this haplotype against bleeding in patients treated with dabigatran or apixaban.

## 1. Introduction

Since their development, almost a decade ago, non-vitamin K oral anticoagulants (NOACs) have swiftly gained their place among the essential drugs in the treatment of non-valvular atrial fibrillation (NVAF), and in recent years, their use has grown significantly in most European countries [1,2,3].

Current guidelines recommend NOACs as first-line treatment in NVAF patients due to the certain clinical benefits they have shown for vitamin-K antagonists (VKA) in the prophylaxis of thromboembolic events, especially strokes [4,5]. 

NOACs have a wide therapeutic window and predictable pharmacokinetics [6], however, they present several disadvantages that make the clinical decision difficult: inability of routine coagulation monitoring, and thus compliance monitoring, marked inter-individual variability in plasma levels, and interactions with certain drugs, some frequently used in the treatment of AF that may increase the risk of bleeding complications, especially in vulnerable patients [7]. The individualization of NOACs doses is an extremely debated topic over recent years. It is assumed that patients who have the greatest benefits from personalized treatment would be those with multiple interfering factors and an inadequate anticoagulant response [8,9].

Dabigatran etexilate, a direct selective thrombin inhibitor, has a reversible linear anticoagulant effect. It is administered at a dose of 150 mg b.i.d or 110 mg b.i.d in patients at a high risk of bleeding [10]. 

Apixaban is a reversible factor Xa inhibitor with a rapid onset of action. Unless patients meet two of the following criteria: age > 80 years, serum creatinine > 1.5 mg/dL and body weight < 60 kg, when 2.5 mg b.i.d is recommended, the standard dose is 5 mg b.i.d. [11]. 

It has been shown that for all NOACs that an inter-individual variability of the response to treatment is caused by different factors [12] of which variations of the genes (“single nucleotide polymorphisms”—SNPs) that encode the enzymes involved in their metabolism could play an important role [13,14,15]. 

An important mechanism of interaction for both dabigatran and apixaban is the significant secretion via P-glycoprotein (P-gp) after intestinal absorption [16]. Both anticoagulants are substrates for P-gp [10,11] and many drugs administered in the AF treatment are P-gp inhibitors (e.g., verapamil, dronedarone, amiodarone, and quinidine) [17]. P-gp presents multiple variations of its function and expression [18]. The *ABCB*_1_ gene is located on chromosome 7q21, has a length of approx. 100 kb and includes 28 exons [18,19]. Several SNPs have been identified in the promoter and exon regions of the *ABCB*_1_ gene, associated with plasma levels of P-gp substrates [19]. However, studies related to the association of *ABCB*_1_ polymorphisms and P-gp expression described in the literature remains controversial and report contradictory results. 

Assuming that the presence of genetic polymorphisms may influence the effects of NOACs treatment on the clinical outcome of NVAF patients, this study aimed to evaluate the association of the *ABCB*_1_ gene SNPs with bleeding in patients from the real-world clinical practice treated with dabigatran and apixaban. 

## 2. Materials and Methods

**Figure 1:**
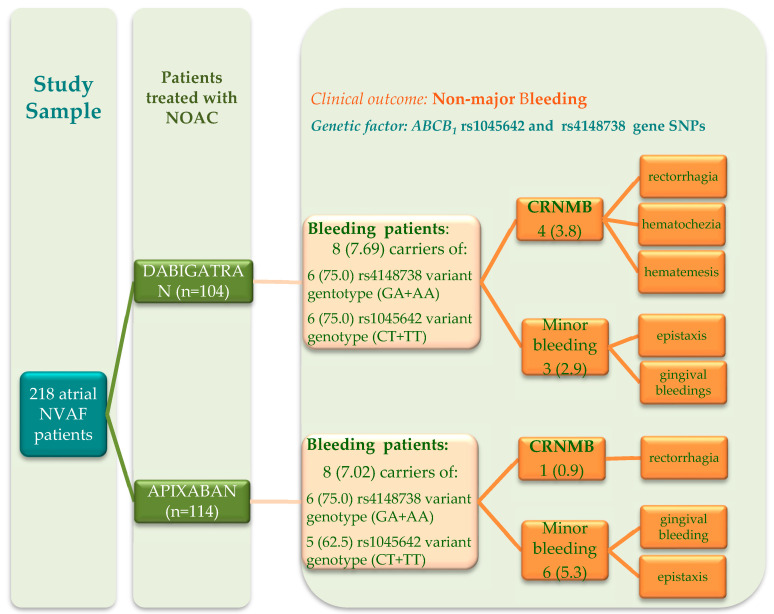
Flowchart diagram: distribution of patients’ data according to the studied clinical outcome and to the *ABCB*_1_ gene SNPs. The data are described as n (%) of patients, where n represents the absolute frequencies and % represent the relative frequencies; two patients had both minor and CRNMB bleeding (1 patient in each of the two subgroups); NVAF = non-valvular atrial fibrillation; NOAC = non-vitamin K oral anticoagulants; CRNMB = clinically relevant non-major bleeding.

This is a single-center prospective observational study conducted from March to December 2019. Consecutive patients of both sexes, older than 18 years old, treated with dabigatran or apixaban for NVAF thromboprophylaxis, regardless of the time of treatment initiation, from the “Niculae Stancioiu” Emergency Heart Institute of Cluj-Napoca, were included (Figure 1). The exclusion criteria were: severe hepatic or renal failure; critical general condition; situations that required treatment discontinuation (surgery, invasive procedures).

For each patient, the anticoagulant treatment and dosing schedule was established by the attending physician.

NVAF has been defined, according to the ESC/EHRA/ACC/AHA recommendations [4,5], as AF of any type (paroxysmal, persistent, or permanent) that occurs in the absence of mechanical valve prostheses and moderate to severe mitral stenosis (usually of rheumatic origin).

Between March and December 2017, patients attended a clinical baseline visit and were evaluated clinically, by ECG, and echocardiography (GE Vivid S6). The individual parameters (age, body mass index), smoking and alcohol consumption habits, comorbidities and concomitant medication (antiplatelet agents, non-steroidal inflammatory drugs, antacids—proton-pump inhibitors, and P-gp inhibitors: amiodarone, quinidine, verapamil, clarithromycin, erythromycin, cyclosporine, itraconazole, ketoconazole, phenobarbital, carbamazepine, phenytoin) were recorded. Also, the additional risk factors for stroke and/or bleeding—age, history of stroke, ejection fraction < 40%, NYHA class > II or symptoms of congestive heart failure in the last six months, diabetes, hypertension, coronary artery disease, peripheral vascular disease, bleeding history or predisposing factors (anemia), unstable INR (time in therapeutic range <60%), and alcohol consumption (more than one or two drinks per day) were registered to calculate the CHA_2_DS_2_-VASc and HASBLED score. 

Chronic kidney disease (CKD) has been defined, according to the KDIGO guidelines, as glomerular filtration rate (GFR) < 90 mL/min/1.73 m^2^ and the presence of structural changes or renal function of at least three months, having an impact on the health condition or GFR < 60 mL/min/1.73 m^2^, without other signs of renal impairment [20]. The GFR was calculated based on the Cockcroft-Gault formula [21].

Major bleeding episodes, according to the criteria of the International Society for Thrombosis and Haemostasis, were defined as clinical emergencies, associated with a decrease in hemoglobin by 2.0 g/dL and/or the need of 2 units of red blood cell transfusions; fatal or symptomatic bleeding in a critical organ (intracranial, pericardial, retroperitoneal) [22]. Clinically relevant non-major bleeding (CRNMB) has been defined as bleeding that does not meet the criteria for major bleeding but requires medical interventions, discontinuation of anticoagulant treatment, or impairment of daily activities [23]. Minor bleeding has been defined as clinically irrelevant bleeding that does not meet the criteria for major bleeding or CRNMB. Gastrointestinal bleeding (GIB) has been defined as bleeding that occurs in any segment of the gastrointestinal tract (esophagus, stomach, small intestine, large intestine, rectum, and anus).

Samples of peripheral venous blood were obtained from all patients for genetic analysis and biological parameters determination (blood count, creatinine, and aminotransferases).

The DNA was extracted in the Department of Medical Genetics Laboratory of “Iuliu Hatieganu” University of Medicine and Pharmacy, Cluj-Napoca, from blood collected into a 2 mL Vacuette^®^ tube K3 EDTA. The commercially available kit was used (Wizard Genomic DNA Purification Kit, Promega, Madison, WI, USA).

Two *ABCB*_1_ SNPs were genotyped: rs4148738 and rs1045642, in all patients, by the real-time PCR technique. For *ABCB*_1_ rs4148738, predesigned TaqMan SNP genotyping assays were used (C_1253813_10).

The other SNP genotyping was performed using custom-designed SNP TaqMan assays. Both Genotyping Assays were acquired from Thermo Fisher Scientific (Waltham, MA, USA) and performed according to manufacturer’s instructions on a QuantStudio 3 Real-Time PCR System machine (Applied Biosystems, Thermo Fisher, Waltham, MA, USA).

The entire cohort of patients was subsequently followed, periodically, until December 2019, by phone or through a clinical visit to the outpatient service of the tertiary cardiology center to assess adherence to treatment and identify the occurrence of possible complications.

The study complied with international ethical standards provided in the Helsinki declaration of human rights and received the ethics committee of the “Niculae Stancioiu” Emergency Heart Institute and the University of Medicine and Pharmacy “Iuliu Haţieganu” Cluj-Napoca approval (no.106/08.03.2017). Informed consent regarding the confidentiality of personal and medical data and agreement to participate in the study was obtained from all patients.

**Statistical Analysis:** Continuous variables with normal probability distribution are presented as mean ± sample standard deviation (SD), while continuous variables with deviation from normal distribution law were represented using median with the interquartile interval (Q1; Q3), where Q1 = lower quartile and Q3 = upper quartile.

The Chi-square or Fisher’s Exact tests were used to assess if demographic and clinical characteristics had different distributions in NVAF patients treated with NOACs (dabigatran and apixaban).

For continuous variables that followed a Gaussian distribution, the homoscedasticity was evaluated by the Bartlett test. The two-sample *t*-test, Welch’s t-test, or Mann–Whitney U test was used to identify statistically significant differences in distributions of continuous variables between different types of NOACs-treated groups.

An exact test of Hardy–Weinberg Equilibrium (HWE) was applied for every SNP in each of the study groups [24].

The association between the odds of bleedings and *ABCB*_1_ SNPs was tested under various inheritance models (codominant, dominant, recessive, and overdominant). Pairwise linkage disequilibrium (LD) parameters (D, D’, and r) were calculated with the genetics R package [25] and haplotype determination was performed by the expectation-maximization (EM) algorithm. Generalized linear regression analysis was performed to test the association of the haplotypes with the bleedings at NOAC patients using the “haplo.stats” R package. The effect size of the haplotypes was estimated by the odds ratios, OR with 95% confidence intervals (95% CI).

All types of statistical analyses were considered significant when *p*-values < 0.05.

Statistical analysis was performed in R software, version 4.0.0 (R Foundation for Statistical Computing, Vienna, Austria).

## 3. Results

### 3.1. Characterization of Patients Treated with NOACs

A total of 218 Caucasian patients were included in the study, receiving dabigatran (n = 104) or apixaban treatment (n = 114).

Demographic, clinical, and echocardiographic characteristics for the entire group of patients treated with NOAC are presented in Table 1. We did not find statistically significant differences in the distribution of demographic characteristics in patients treated with dabigatran and those treated with apixaban (*p* > 0.05). We did not find a statistically significant difference in CHA_2_DS_2_-VASc (*p* = 0.975) and HAS-BLED scores (*p* = 0.206), as well as in echocardiographic parameters values in patients treated with dabigatran vs. those receiving apixaban.

Patients treated with apixaban had, on average, lower creatinine clearance values than patients treated with dabigatran (77.58 ± 25.98 mL/min/1.72 m^2^ vs. 81.57 ± 28.53 mL/min/1.72 m^2^) but without statistical significance (*p* = 0.281). Unlike apixaban-treated patients, the majority of dabigatran-treated patients (61.54%) previously had VKA treatment. The percentage of patients receiving P-gp inhibitors (amiodarone, quinidine, verapamil, clarithromycin, and carbamazepine) and antiplatelet agents was similar between the two groups.

### 3.2. The Association between ABCB_1_ SNP and Odds of Bleedings

The polymorphisms’ distribution on the entire group of patients treated with NOACs and in both subgroups, respected the Hardy–Weinberg equilibrium’s conditions (*p* > 0.05).

The frequency of ABCB_1_ gene polymorphisms is described in Table 2.

For both ABCB_1_ polymorphisms, heterozygous patients predominated both in the whole studied group and within the subgroups (Table 2). Among the dabigatran-treated patients that had experienced bleedings, 75% were carriers of rs4148738 variant genotype (GA+AA) and 75% were carriers of rs1045642 variant genotype (CT+TT). In patients treated with apixaban who had bleedings, 75% were carriers of the rs4148738 variant genotype (GA+AA, both AA carriers presented rectorrhagia) and 62.5% were carriers of the rs1045642 variant genotype (CT+TT).

The logistic tested models based on the four genetic models (codominant, dominant, recessive, and overdominant) revealed no significant association between the two ABCB_1_ SNPs and the risk of bleeding (Table 3).

Although we noticed that the variant genotype of ABCB_1_ rs1045642 C > T was associated with decreased odds of bleedings (adjusted OR > 1) while ABCB_1_ rs4148738 G>A was associated with increased odds of bleedings (adjusted OR > 1), the associations were of no statistical significance (*p* > 0.05).

Bleeding complications occurred in 16 NOACs-treated patients (7.34%). Most of them were CRNMB and required temporary cessation of treatment (48–72 h). In patients treated with dabigatran they were represented by five episodes of rectorrhagia; two of hematochezia and one episode of hematemesis. The last case occurred after the association of NSAIDs and the patient was subsequently prescribed a low dose of apixaban. In the apixaban-treated group during the follow-up we identified three episodes of rectorrhagia; two patients received concomitant antiplatelet and NSAIDs therapy. In all patients, the treatment with NOACs was resumed, with the same doses, on the attending physician recommendation, after seeing a gastroenterologist. Also, several minor bleeding episodes were recorded: two gingival bleedings and four episodes of epistaxis in patients treated with dabigatran and three episodes of epistaxis and four gingival bleeding in those treated with apixaban. In two patients these minor episodes were concomitant with CRNMB.

Concerning the traditional known factors for the bleeding, age greater than 70 years and stroke/TIA showed a tendency toward statistical significance (*p* < 0.10) (Table 4).

### 3.3. Haplotype Analysis

The ABCB_1_ haplotype analysis revealed that the haplotype set was covered by four haplotypes in NOACs patients with and without bleedings (Table 5). Taking the common CG haplotype as reference (OR = 1), the results of the haplotype-based generalized linear model (logistic regression) found no significant association of haplotypes with bleedings in NOACs-treated patients (*p* > 0.05).

## 4. Discussion

Although there are clear recommendations regarding the administration of NOACs’ type and doses [4,5], in clinical practice the monitoring of the anticoagulation level, an accurate classification in risk classes, and treatment individualization may be of vital importance in certain patients.

Despite being easier to use than VKA, NOACs retain some of the drawbacks of any anticoagulant treatment. Firstly, their short half-life increases the risk of thromboembolic events in patients with reduced compliance. However, the most important issue encountered in clinical practice is the approach of bleeding complications [27,28] in the absence of routine laboratory tests to determine the anticoagulant level and with expensive and less accessible reversing agents (only recently approved) [29,30].

Also, a considerable number of NOACs-treated patients are exposed to invasive procedures or emergency surgery each year. For example, in the RE-LY trial (Randomized Evaluation of Long-Term Anticoagulation Therapy), during the two years of follow-up, 25% of dabigatran-treated patients underwent at least one intervention that required discontinuation of NOAC (7.8% were emergency interventions) [31].

The interest in the role of pharmacogenomics in cardiovascular disorders is currently increasing [32]. Identifying the genetic variants that predict the clinical outcome of anticoagulant treatment facilitates the clinical decision in choosing the most favorable type and dose of NOACs [32,33]. Genotyping is very useful in personalizing treatment, especially in high-risk patients [32].

The routine activity in our tertiary emergency center for cardiovascular diseases involves numerous invasive procedures with an increased risk of bleeding. This is why we chose to analyze the influence of *ABCB*_1_ gene variations on the first NOACs used in patients with NVAF: dabigatran and apixaban, in a group of patients recruited from the real-world clinical practice. These two NOACs have different mechanisms of action and pharmacokinetics, but the plasma half-life is similar (10 to 12 h). For this reason, both are administered in fixed doses, twice a day. In our previously published studies, we evaluated plasma levels and genotype-phenotype correlations of *ABCB*_1_ SNPs (which to our knowledge are determined for the first time in a Romanian population sample), in patients treated with apixaban [34] and dabigatran, respectively [35]. The present study aimed to investigate, in a larger number of patients, whether the *ABCB*_1_ polymorphisms rs4148738 and rs1045642 have a significant impact on the clinical outcome of patients during the two NOACs administration. The two *ABCB*_1_ SNPs are among the very few investigated so far in relation to NOACs’ pharmacokinetics in the only genomic association study (GWAS) (14) and small individual studies [15,36,37,38,39].

Heterozygous patients predominated in our studied group for both *ABCB*_1_ polymorphisms. This finding is consistent with the genotype distribution obtained in other studies with Caucasian subjects [36,37,38,39].

In the present study, bleedings appeared in 7.34% NOAC-treated patients. Phase III randomized trials comparing NOACs with warfarin and real-world studies [40,41] estimated the bleeding risk at 1 to 3% per year, and discontinuation of NOACs was less prevalent than with VKA [41]. As well, both individual studies and meta-analyses have shown that NOACs are associated with a lower incidence of fatal bleedings, lower in-hospital mortality, and a better evolution of patients suffering from bleedings, even intracranial ones [27]. Moreover, patients with GIB treated with NOACs had a shorter hospital stay and required less endoscopy than those treated with VKA [27,42].

During the follow-up period, we did not register major bleedings, only CRNMB and minor bleeding episodes, easily managed by gastroenterologists and treating cardiologists. Generally, non-major bleedings were more common than the major ones in NOACs randomized trials [43] and real-world studies [40,41]. As shown before, also in our study, in the vast majority, predominated lower GIB [44] possible by incomplete absorption of the active substance in the upper tract and increased NOACs availability in the lower tract [45]. NOACs, unlike VKA and other parenteral anticoagulants, are thought to exhibit intraluminal anticoagulant activity through their incomplete absorption into the digestive mucosa [42].

Among the NOACs treated patients in our study that have experienced bleedings, 75% were carriers of the rs4148738 variant genotype (GA+AA) and 68.75% were carriers of the rs1045642 variant genotype (CT+TT). Similar results were reported in the article published by Sychev et al. [37], in which the presence of the rs1045642 (C > T)’s variant genotype was observed in a higher percentage in patients who presented bleeding episodes, without reaching statistical significance.

To evaluate the possible association between *ABCB*_1_ SNPs and the bleeding risk we tested four inheritance models (codominant, dominant, recessive, and overdominant), but they revealed no significant results, possibly because our patients experienced very few side effects during NOACs treatment. The results obtained for the two SNPs were divergent: the variant genotype of *ABCB*_1_ rs4148738 (G > A) was associated with increased odds of bleeding, while rs1045642 (C > T) was associated with decreased odds of bleeding.

In addition, we tested the association between the type of bleeding (minor bleeding, CRNMB, and both) and *ABCB*_1_ genotypes under the dominant genetic model due to the small number of NOAC-treated patients. We found no significant associations between the type of bleeding and rs4148738 variant genotype GA+AA (Fisher’s exact test, *p* = 0.454) or rs1045642 variant genotype CT+TT (Fisher’s exact test, *p* = 0.800).

Our results regarding haplotype analysis revealed that, among the four two-locus haplotypes, TA and CA haplotypes had the highest frequency in NOACs-treated patients with bleedings, involving a possible positive association with the susceptibility of bleeding complications (OR = 1.04 and OR = 1.91, respectively). However, in the present study, we did not reach statistical significance, possibly because of the small number of cases with the outcome of interest.

Also, in our sample, there was a trend toward statistical significance for the association between TG haplotype and bleedings (*p* = 0.092). The haplotype frequency was higher in patients without bleedings than in those with bleedings, implying a possible protective role of this haplotype against bleeding in NOACs-treated patients.

In other previously published studies, the results obtained also did not reveal significant associations between *ABCB*_1_ SNPs and the clinical events. Furthermore, data on the relationship between these SNPs and the NOACs plasma levels were conflicting. In most studies, *ABCB*_1_ rs4148738 (G>A) variant alleles carriers had higher NOACs plasma concentrations [14,38,39]. This trend was also observed in our previous study [34] and could be in accordance with the increased bleeding odds (above mentioned), but the small number of clinical events, as well as the insufficient number of patients studied in most individual research, does not allow us to make an undeniable statement.

The *ABCB*_1_ rs1045642 (C > T) has been widely studied and researches have shown that it affects the function of P-gp [19]. The minor allele carriers had higher NOACs plasma concentrations without statistical significance [15,37] or did not present any relationship with their levels [39,46]. Furthermore, the *ABCB*_1_ gene encodes the P-gp transmembrane transporter, which plays a protective role by limiting the absorption of its substrates from the digestive tract and promoting their elimination by the liver and kidneys [19]. The loss of its function, due to genetic variations, has been shown to alter the substrates’ pharmacokinetic profiles [47], usually enhancing their plasma levels. For this reason, also in the case of this SNP, the bleeding risk should increase.

We have also analyzed the traditional known risk factors for bleeding. Only “age greater than 70 years” is itself a risk factor and stroke/TIA showed a tendency toward statistical significance.

In AF patients treated with anticoagulants, the absolute risk of bleeding ranged from 1.3 to 7.2%, being higher in patients over 75 years of age, with a history of stroke and multiple associated diseases (hypertension, diabetes) [48,49]. Observational studies [50] and meta-analyses showed a higher tendency to bleed in elderly patients treated with NOACs vs. warfarin [51]. A direct comparative study between NOACs also confirmed that the risk of bleeding increases with age [49]. Also, in real-world NOACs studies history of GIB, alcohol consumption, and renal and hepatic dysfunction resulted in an increase in the incidence of GIB compared to that reported in randomized trials [40]. The concomitant administration of several drugs whose metabolic pathways are common with those of NOACs (P-gp or CYP_3_A_4_ inhibitors) increases plasma levels and the risk of bleeding [17].

In our study, the demographic, clinical, echocardiographic characteristics, and the CHA_2_DS_2_-VASc and HAS-BLED scores were similar in the two subgroups treated with dabigatran or apixaban. In the entire studied group, the patients who presented bleedings were older, but none of them received treatment with P-gp inhibitors, had gastrointestinal disorders or history of previous bleeding episodes.

The fact that no major bleedings were observed during the patients’ follow-up, in the present study, in a two-year follow-up period, is consistent with the recently published data from the GLORIA-AF registry which showed that NOACs administration in patients with NVAF causes a low incidence of stroke, major bleeding, and myocardial infarction in current clinical practice, confirming the safety and efficacy of these anticoagulants [52].

The present study had several limitations: first, the frequency of bleedings was lower; that led to no significant *p*-values in the statistical analysis of genotypes and haplotypes of the *ABCB*_1_ gene. Second, the analysis of the multivariate effect of genotype and haplotype on the outcome of interest was not performed because of the reduced number of bleeding complications so we limited to estimate the univariate effect of each of the SNPs.

Although we aimed to study the NOACs-treated patients’ outcomes in real-world clinical practice conditions, in the end, our study group consisted of a homogeneous cohort of patients with similar characteristics to those in randomized trials. In our country, NOACs, which are expensive, have not been reimbursed by national insurances for a long time. As a result, a limited number of patients, with average incomes, could afford to follow this treatment. Generally, these patients respect medical recommendations and have adequate treatment adherence. Furthermore, clinicians are reluctant to prescribe these drugs to frail patients or those at high risk of bleeding due to the lack of routine monitoring of the anticoagulant effect; they strictly follow the guidelines recommendations regarding the dosing regimen and monitor more often NOACs-treated patients. This unwillingness to prescribe NOACs is also reported in real-world studies in recent years [53,54]. Periodic monitoring of patients greatly improves treatment compliance. Patients included in the major clinical trials were closely monitored, which also contributed to the very good results obtained with NOACs [55]. It is also important to choose the optimal anticoagulant and dose and to periodically review the treatment associated with interaction potential and the renal function.

Even minor or non-major bleeding episodes are important in the evolution of patients because they affect their quality of life [7] and are often followed by discontinuation of anticoagulant therapy which in turn increases the risk of mortality. In real-world studies, it was estimated that less than 50% of patients resume anticoagulant treatment after a bleeding episode [53,54].

GIB is one of the key issues to consider in evaluating patients to which it is desired the anticoagulant treatment initiation, especially in the elderly, because GIB is associated with a poor prognosis and can significantly affect their quality of life. The more frequently NOACs are prescribed, the more important becomes the bleeding risk assessment in clinical practice.

Creating and validating specific NOACs standard for long-term estimation of bleeding risk can help clinicians individualize the therapy of their patients. It remains to be established in much larger studies whether some gene combinations increase the risk of bleeding in patients treated with NOACs.

## 5. Conclusions

The present study found no significant associations between variant genotypes of *ABCB*_1_ rs1045642(C > T) and *ABCB*_1_ rs4148738 (G > A) with non-major bleeding risk in NOACs-treated patients. In our sample of patients treated with dabigatran or apixaban, we found a trend of association between TG haplotype with bleeding risk, implying a protective role of this haplotype against bleeding in patients treated with dabigatran or apixaban. Future studies with a larger sample size are needed to confirm these results.

## Figures and Tables

**Table 1:** Baseline characteristics of the patients.

Demographic and Clinical Variables	NOAC Therapy (n = 218)	DABIGATRAN (n_1_ = 104)	APIXABAN (n_2_ = 114)	*p*-Value
Age (years), mean ± SD	70.94 ± 9.04	70.89 ± 8.85	70.97 ± 9.24	0.949
Male, f_a_ (%)	113 (51.83)	55 (52.88)	58 (50.88)	0.767
AF type, f_a_ (%)				0.980
paroxysmal	74 (33.94)	36 (34.62)	38 (33.33)	
persistent	51 (23.39)	24 (23.08)	27 (23.68)	
permanent	93 (42.66)	44 (42.31)	49 (42.98)	
BMI (kg/m^2^), mean ± SD	28.06 ± 4.32	28.83 ± 4.11	27.35 ± 4.41	0.012 *
CHA_2_DS_2_-VASc score ≥ 2	199 (91.28)	95 (91.35)	104 (91.23)	0.975
HAS-BLED score ≥ 3	29 (13.30)	17 (16.35)	12 (10.53)	0.206
CrCl ml/min/1.72 m^2^, mean ± SD	79.48 ± 27.24	81.57 ± 28.53	77.58 ± 25.98	0.281
LADi (mm/ m^2^), median (Q1; Q3)	22.78 (20.72; 25.75)	22.12 (20.08; 25.02)	23.38 (21.10; 26.25)	0.016 *
LAVi (ml/ m^2^), median (Q1; Q3)	33.39 (30.55; 40.01)	33.44 (30.20; 38.92)	35.57 (31.62; 40.37)	0.184
Prior VKA, f_a_ (%)	102 (46.79)	64 (61.54)	38 (33.33)	<0.0001 **
P-gp inhibitors, f_a_ (%)	54 (24.77)	24 (23.08)	30 (26.32)	0.580
Antiplatelet. f_a_ (%)	33 (15.14)	14 (13.46)	19 (16.67)	0.510
NSAIDs, f_a_ (%)	16 (7.34)	11 (10.58)	5 (4.39)	0.080
Bleeding, f_a_ (%)	16 (7.34)	8 (7.69)	8 (7.02)	0.849

SD—standard deviation; f_a_—absolute frequency; Q1—first quartile; Q3—third quartile; estimated significance level (*p*) obtained from Chi-square tests or Fisher’s exact test in testing the differences between the qualitative variables distributions or the t-Student test and the non-parametric Mann–Whitney test in testing the differences between the quantitative variables distributions in NVAF patients treated with Dabigatran versus Apixaban;* *p* < 0.05; ** *p* < 0.01; AF—atrial fibrillation; BMI—body mass index; CrCl—creatinine clearance; LADi—left atrial indexed diameter; LAVi—left atrial indexed volume; VKA—vitamin-K antagonists; P-gp—P glycoprotein, NSAIDs—nonsteroidal anti-inflammatory drugs.

**Table 2:** *ABCB*_1_ genotypes distribution according to the presence of bleeding in patients treated with dabigatran and apixaban, respectively.

SNPs	NOAC Therapy	DABIGATRAN				APIXABAN			
	(n = 218)	Total (n_1_ = 104)	Bleeding (n_1_ = 8)	No Bleeding (n_2_ = 96)	*p*-Value	Total (n_2_ = 114)	Bleeding (n_1_ = 8)	No Bleeding (n_2_ = 106)	*p*-Value
*ABCB*_1_ rs1045642 C > T MAF CEU* = 43.4		p_HWE_ = 0.230				p_HWE_ = 0.710			
MAF (95% CI)	46.56 (41.80; 51.37)	43.27 (36.44; 50.30)	43.75 (19.75; 70.12)	43.23 (36.12; 50.56)	-	49.56 (42.90; 56.39)	37.50 (15.20; 64.56)	50.47 (43.54; 7.39)	-
Genotype					0.965				0.325
CC	60 (27.52)	30 (28.84)	2 (25)	28 (29.16)		30 (26.31)	3 (37.5)	27 (25.47)	
CT	113 (51.83)	58 (55.76)	5 (62.5)	53 (55.2)		55 (48.24)	4 (50)	51 (48.11)	
TT	45 (20.64)	16 (15.38)	1 (12.5)	15 (15.62)		29 (25.43)	1 (12.5)	28 (26.4)	
*ABCB*_1_ rs4148738 G > A MAF CEU* = 46.0		p_HWE_ = 0.160				p_HWE_ = 0.850			
MAF (95% CI)	43.35 (38.64; 48.15)	41.35 (34.58; 48.36)	50.00 (24.65; 75.35)	40.63 (33.61; 47.93)	-	45.18 (38.60; 1.88)	50.00 (24.65; 75.35)	44.81 (37.99; 51.77)	-
Genotype					0.428				0.691
GG	67 (30.73)	32 (30.76)	2 (25)	30 (31.25)		35 (30.70)	2 (25)	33 (31.13)	
GA	113 (51.83)	58 (55.76)	4 (50)	54 (56.25)		55 (48.24)	4 (50)	51 (48.11)	
AA	38 (17.43)	14 (13.46)	2 (25)	12 (12.5)		24 (21.05)	2 (25)	22 (20.75)	

SNP—single nucleotide polymorphism; MAF CEU*—Utah residence with northern and western European ancestry [26]; MAF—minor allele frequency; CI—confidence interval; the data were presented as absolute frequencies and relative frequencies (%); estimated significance level (*p*) was obtained by applying the Cochran–Armitage test for differences in genotype frequencies in bleeding vs. no bleeding patients; p_HWE-_ *p*-value obtained from the verifying Hardy–Weinberg equilibrium test in patients treated with dabigatran and apixaban, respectively.

**Table 3:** The association of the two *ABCB*_1_ polymorphisms with the bleeding odds in NOAC—treated patients (dabigatran or apixaban).

SNPs	Tested Genetic Models	Genotype	Adjusted OR (95% CI)	*p*-Value
*ABCB*_1_ rs1045642 C > T	Codominant	CC	Reference	0.685
		CT	0.95 (0.30; 2.98)	
		TT	0.52 (0.09; 2.80)	
	Dominant	CC	Reference	0.736
		CT+TT	0.83 (0.27; 2.45)	
	Recessive	CC+CT	Reference	0.386
		TT	0.53 (0.12; 2.41)	
	Overdominant	TT+CC	Reference	0.723
		CT	1.20 (0.43; 3.37)	
*ABCB*_1_ rs4148738 G > A	Codominant	GG	Reference	0.688
		GA	1.19 (0.35; 4.13)	
		AA	1.88 (0.44; 8.04)	
	Dominant	GG	Reference	0.599
		GA +AA	1.36 (0.42; 4.38)	
	Recessive	GG + GA	Reference	0.414
		AA	1.68 (0.51; 5.57)	
	Overdominant	GG+ AA	Reference	0.867
		GA	0.92 (0.33; 2.54)	

CI—confidence interval; odds ratio adjusted for the effect of NOAC treatment type (dabigatran or apixaban).

**Table 4:** Univariate Logistic Regression Model for bleedings in NOAC-treated patients.

	Bleeding (n_1_ = 16)	No Bleeding (n_2_ = 202)	Unadjusted OR (95% CI)	*p*-Value
Age (>70 years vs. ≤ 70)	12 (75.00)	104 (51.49)	2.83 (0.95; 10.38)	0.080
Sex (male vs. female)	6 (37.50)	107 (53.00)	0.53 (0.18; 1.49)	0.239
Diabetes mellitus (yes vs. no)	7 (43.75)	50 (24.75)	2.36 (0.81; 6.67)	0.104
Hypertension (yes vs. no)	14 (87.50)	167 (82.67)	1.47 (0.39; 9.61)	0.622
Stroke/TIA history (yes vs. no)	5 (31.32)	28 (13.86)	2.82 (0.84; 8.42)	0.072
Ischemic heart disease (yes vs. no)	3 (18.75)	45 (22.28)	0.81 (0.18; 2.63)	0.744
Peripheral vascular disease (yes vs. no)	2 (12.50)	22 (10.89)	1.17 (0.18; 4.56)	0.843
Heart failure (yes vs. no)	1 (6.25)	32 (15.84)	0.35 (0.02; 1.84)	0.323
Chronic kidney disease (yes vs. no)	4 (25.00)	53 (26.24)	0.94 (0.25; 2.82)	0.914
Gastritis/peptic ulcer (yes vs. no)	0 (0.00)	6 (3.0)	ND	0.993
Smoking ^#^ (yes vs. no)	2 (12.50)	18 (9.5)	1.37 (0.20; 5.43)	0.696
Alcohol consumption ^#^(yes vs. no)	2 (12.50)	8 (4.2)	3.25 (0.46; 14.58)	0.159

^#^ univariate logistic regression was performed on n = 206 cases and 107 cases, respectively; ND—cannot be determined due to lack of cases; TIA—transient ischemic attack.

**Table 5:** Haplotype results.

Haplotypes	Haplotype-Frequencies (%) in NOAC Patients			Hap-Score Statistics ^(a)^	p ^(b)^	Unadjusted OR ^(c)^, 95% CI (Lower; Upper Limit)
	Overall Sample	with Bleedings	without Bleedings			
C-G	48.41	50.00	48.32	0.13	0.894	1.00 (Reference)
*T-A*	38.32	40.63	38.17	0.23	0.816	1.04 (0.47; 2.31)
*T-G*	8.25	0.00	8.86	−1.68	0.092	<0.00001
*C-A*	5.03	9.36	4.65	1.33	0.183	1.91 (0.53; 6.93)

Note. Haplotypes estimated from the two gene variants are ordered according to the estimated frequency in studied sample; ^(a)^ haplotype score for the haplotype; ^(b)^ p-values obtained from the Score test; ^(c)^ odds ratios (OR) estimated from haplotype-based generalized linear model without covariates.
